# Corelation between Beck Depression Inventory and stress coping strategies scale on parents of children with Spina Bifida

**DOI:** 10.1192/j.eurpsy.2023.1232

**Published:** 2023-07-19

**Authors:** V. Ozer, P. Ulual, G. Orhaner, I. Alataş, R. Çetiner, O. Güçlü

**Affiliations:** 1 Istanbul Basaksehir Cam ve Sakura Sehir Hastanesi; 2 Yeşilay Neuropsychiatry department; 3 İstanbul Bilim Üniversitesi; 4 İstanbul Eğitim ve Araştırma Hastanesi, İstanbul, Türkiye

## Abstract

**Introduction:**

Spina Bifida(SB), in other terms called spine openness, is a prenatal disease occur due to improper closure of the spine of the fetus during the first months of the pregnancy.

**Objectives:**

Having a disabled child or to observe deficiency in a child regardless of its level is a highly stressful situation for the families. To take care of such children causes an emotional and physical burden on the parents. Thus, this leads to an increase in the level of depression and anxiety on these individuals, causes health related problems and an increase in the drug usage.

**Methods:**

Beck depression Inventory and stress coping strategies scale have been applied to 66 parents consisting of 39 female and 27 males. The cut-off scores for Beck Depression Inventory were 1-10 for normal, 11-16 for mild mood disturbance, 17-20 for borderline clinical depression, 21-30 for moderate depression, 31-40 for severe depression. For stress coping strategies scale higher scores correlated with the intensity of coping mechanisms listed on the scale.

**Results:**

Acquired data from 66 parents show a positive correlation between Beck Depression Inventory and Stress coping strategies scale

**Conclusions:**

The lower the individuals BDI scores, the lower the stress coping strategies scale scores were meaning that the intensity of depression correlates with the level of coping mechanisms. This suggests that parents of patients with diseases like SB should get the needed psychiatric help and supportive care during the course of treatment.

**Disclosure of Interest:**

None Declared

